# Reduced paxillin expression contributes to the antimetastatic effect of 4-hydroxycoumarin on B16-F10 melanoma cells

**DOI:** 10.1186/1475-2867-8-8

**Published:** 2008-05-20

**Authors:** Marco A Velasco-Velázquez, Nohemí Salinas-Jazmín, Nicandro Mendoza-Patiño, Juan J Mandoki

**Affiliations:** 1 Departamento de Farmacología, Facultad de Medicina, Universidad Nacional Autónoma de México. Apdo. Postal 70-297, Ciudad Universitaria, México D.F. 04510, México

## Abstract

**Background:**

4-Hydroxycoumarin (4-HC) is a coumarin that lacks anticoagulant activity. 4-HC affects the cytoskeletal stability and decreases cell adhesion and motility of the melanoma cell line B16-F10. Together with integrins and other cytoskeletal proteins, paxillin participates in the regulation of cell adhesion and motility, acting as an adapter protein at focal adhesions. The present study determined the participation of paxillin in the reported effects of 4-HC and analyzed the role of paxillin in the formation of melanoma metastases.

**Results:**

4-HC decreased protein and mRNA levels of α- and β-paxillin isoforms in B16-F10 cells. Paxillin downregulation correlated with an inadequate translocation of paxillin to focal adhesions and a reduced phosphotyr^118^-paxillin pool. Consequently, 4-HC altered paxillin-mediated signaling, decreasing the phosphorylation of FAK and the level of GTP-bound Rac-1. These results partially explain the mechanism of the previously reported effects of 4-HC. Additionally, we studied the effect of 4-HC on metastatic potential of B16-F10 cells through experimental metastasis assays. *In vitro *treatment of cells with 4-HC inhibited their capability to originate pulmonary metastases. 4-HC did not affect cell proliferation or survival, demonstrating that its antimetastatic effect is unrelated to changes on cell viability. We also studied the importance of paxillin in metastasis by transfecting melanoma cells with paxillin-siRNA. Transfection produced a modest reduction on metastatic potential, indicating that: i) paxillin plays a role as inducer of melanoma metastasis; and ii) paxillin downregulation is not sufficient to explain the antimetastatic effect of 4-HC. Therefore, we evaluated other changes in gene expression by differential display RT-PCR analysis. Treatment with 4-HC produced a downregulation of Adhesion Regulating Molecule-1 (ARM-1), which correlated with a decreased adhesion of melanoma cells to lung slides.

**Conclusion:**

This study shows that reduced paxillin expression is associated with the impaired cell adhesion and motility seen in 4-HC-treated cells and partially contributes to the antimetastatic effect of 4-HC. In contrast, the role of ARM-1 reduced expression in the effects of 4-HC is still to be clarified. The antimetastatic effect of 4-HC suggests that this compound, or others with similar mode of action, might be useful for the development of adjuvant therapies for melanoma.

## Background

Formation of metastases is still the leading cause of death in advanced melanoma patients, regardless the numerous innovations on the treatment of the disease. Hence, the search of therapeutic agents that can inhibit metastasis is crucial for improving the management of melanoma.

The production of metastases is a highly complex process by which some cancer cells move away from the primary tumor and colonize other organs. This process requires phenotypical changes that allow cancer cells to migrate, survive in the blood circulation, extravasate, and proliferate in a tissue with a different microenvironment [1]. Along the metastatic cascade, cell adhesion is an essential process [2]. Cell adhesion mediated by integrin receptors drives the formation of focal adhesions, which are multimolecular structures that enable cells to firmly adhere. Additionally, focal adhesions constitute important signaling centers that regulate the reorganization of the cytoskeleton needed for spreading and motility [3,4]. These functions are critical in the acquisition of the ability of cancer cells to invade distant tissues [2]; consequently, the focal adhesion molecules have been proposed as pharmacological targets for decreasing invasiveness of cancer cells [5,6].

Paxillin is a multidomain adapter protein that participates in linking scaffolding and in signaling at focal adhesions [7]. The structural features of paxillin (reviewed by Brown and Turner [7]) allow it to interact with different signaling proteins, such as FAK, Src, Crk, Csk, p120 RasGAP, and PTP-PEST [7,8]. Thus, paxillin has been implicated in the regulation of diverse cellular events, including adhesion [9,10], spreading [11], and motility [11,12].

In mouse, two isoforms of paxillin are generated by alternative splicing, with molecular weights of 68 (α) and 70 KDa (β) [13,14]. Even when both isoforms may share the same functions [14], the β isoform has been implicated in transformation and malignancy [13]. Both paxillin isoforms contain critical phosphorylation sites at tyrosines 31 and 118 [7,12]. The phosphorylation of these two tyrosines by FAK and Src regulates the paxillin turnover in focal adhesions [15-17] and generate docking sites for other molecules that participate in the rearrangement of the actin cytoskeleton [7,16-19]. Then, tyrosine phosphorylation of paxillin and its localization into focal adhesions are necessary for the adequate control of adhesion and motility [12,17-19].

In cancer cells, overexpression and increased tyrosine phosphorylation of paxillin have been reported. For example, paxillin overexpression stimulates the adhesion of squamous carcinoma cells to collagen [20] as well as their migration [21]. Paxillin is also overexpressed in highly metastatic human osteosarcoma [22] and renal carcinoma cell lines [23]. Similarly, levels of phospho-paxillin are much higher in melanoma cell lines than in melanocytes [24]. These data suggest that paxillin plays a role in the acquisition and maintenance of a malignant phenotype.

4-Hydroxycoumarin (4-HC) is a simple coumarin used as precursor for the synthesis of anticoagulant drugs and rodenticides that are 3-substituted-4-hydroxicoumarins; however, 4-HC lacks of anticoagulant activity [25]. Previously, we have provided evidence that 4-HC affects the stability of the actin cytoskeleton on the melanoma cell line B16-F10, impairing the formation of stress fibers and lamellipodia [26]. These effects correlate with reductions in cell adhesion to extracellular matrix proteins and inhibition of motility [26]. The key role of paxillin in the regulation of cytoskeletal rearrangements, adhesion, and motility led us to the proposal that paxillin may be involved in the reported effects produced by 4-HC. Therefore, we analyzed the effects of 4-HC on paxillin expression and paxillin-mediated signaling. Additionally, we evaluated the metastatic potential of B16-F10 cells treated with 4-HC, and studied the role of paxillin in metastasis by blocking its expression with siRNA. Finally, we performed differential display RT-PCR assays in order to identify other proteins that participate in the effects of 4-HC.

## Methods

### Materials

The murine melanoma cell line B16-F10 was purchased from the American Type Culture Collection (Manassas, VA, USA). C57BL/6 mice (Harlan, Mexico City, Mexico) were used in this study. The experiments with mice were conducted in accordance with the Guide for the Care and Use of Laboratory Animals as adopted and promulgated by the Declaration of Helsinki.

4-Hydroxycoumarin (4-Hydroxy-2H-1-benzopyran-2-one [cat. no. H23805]) and its vehicle, ethanol, were purchased from Sigma (St. Louis, MO, USA). Antibodies against paxillin (sc-5574 rabbit polyclonal), β-tubulin (D-10 mouse monoclonal), FAK (H-1 mouse monoclonal), and phosphotyrosine (PY20 mouse monoclonal) as well as siRNAs were obtained from Santa Cruz Biotechnology (Santa Cruz, CA, USA). Control siRNA (sc-37007) is a non-targeting 20–25 nt siRNA that will not lead to the specific degradation of any known cellular mRNA, while paxillin siRNA (sc-36197) is a pool of 3 target-specific 20–25 nt siRNAs.

### Cell culture and treatments

B16-F10 cells were routinely cultured at 37°C in a humid 5% CO_2 _atmosphere, using RPMI-1640 containing 10% fetal bovine serum (FBS). For all experiments cells were seeded at a density of 3 × 10^4 ^cells/cm^2^. In experiments with 4-HC, cells were incubated overnight and then exposed for 24 h to 500 μM 4-HC (dissolved in ethanol) or 0.75% ethanol (control) in serum-free medium. The concentration and exposure time used here were previously reported [26], and are those that induce changes in actin cytoskeleton, impairing cell adhesion and motility.

Transfection with siRNA (60 pmols) was carried out with Lipofectamine 2000 (Invitrogen, Rockville, MD, USA) according to the procedure recommended by the manufacturer.

### Analysis of paxillin expression and phosphorylation

Treated cells were washed twice with ice-cold PBS and lysed in cold lysis buffer [50 mM Tris (pH 8.0), 150 mM NaCl, 1% NP-40, 0.5% deoxycholate, 0.1% SDS] containing a protease inhibitor cocktail (Roche, Indianapolis, IN, USA). Samples containing 60 μg of total protein were separated by SDS-PAGE and transblotted onto PVDF membranes. The membranes were blocked and then probed with anti-paxillin or with anti-phosphotyr^118^-paxillin antibodies. On both cases the membranes were stripped and reprobed with anti-β-tubulin antibody. After incubation with the corresponding secondary antibody, the immunoreactive bands were visualized by chemiluminiscence and a densitometric analysis was carried out using ImageJ NIH software [27].

### Immunoflurescence

Cellular localization of paxillin was evaluated on cells seeded on Labtek chambers (Nunc, Rochester, NY, USA). After treatment, cells were fixed with 4% formaldehyde in PBS and permeated during 4 min at room temperature with 0.1% Triton X-100 diluted in PBS. Then, paxillin was labeled using anti-paxillin antibody followed by a secondary antibody conjugated with Alexa-546 (Molecular Probes, Eugene, OR, USA). After extensive washing, the slides were mounted and analyzed with a Nikon epifluorescence microscope (Melville, NY, USA).

### Reverse transcription-polymerase chain reaction (RT-PCR)

Total RNA was extracted using Trizol reagent (Invitrogen) according to the manufacturer's instructions and quantified spectrophotometrically at 260 nm. Synthesis of cDNA was carried out using Super-Script reverse transcriptase (Invitrogen) and oligo-dT as primer. Semiquantitative PCR was performed in 50 μl of a reaction mixture containing 1× PCR buffer, 2.5 mM MgCl_2_, 0.2 mM dNTPs, 1·U Ampli*Taq *DNA polymerase (Invitrogen), 0.2 μM of each primer, and cDNA obtained from 175 ng of total RNA. Reactions were cycled 30 times through 30 s at 94°C, 60 s at 55°C, and 60 s at 72°C. The paxillin primers used were previously reported by Mazaki *et. al*. [14] and are as follows: pan-paxillin: sense 5'-aacaagcagaagtcagcagagcc-3', antisense 5'-ctagcttgttcaggtcggac-3' (amplicon length: 582 bp for α isoform and 684 bp for β isoform); β-paxillin: same sense primer than for pan-paxillin, antisense 5'-ctctccatccactctctgtt-3' (503 bp). The GAPDH primers [28] were: sense 5'-accacagtccatgccatcac-3', antisense 5'-tccaccaccctgttgctgta-3' (452 bp). PCR products were resolved by 2% agarose gel electrophoresis and stained with ethidium bromide. Negative controls lacking reverse transcriptase were run in parallel to confirm that samples were not contaminated with genomic DNA.

Real time PCR for ARM-1 was performed in a GeneAmp 5700 Sequence Detection System (Applied Biosystems, Foster City, CA, USA) as described previously [29 javascript:popRef('b12')]. Briefly, 0.5× of SYBR Green I (Molecular Probes, Eugene, OR, USA) was added to the reaction mixture described above. The primers for amplification of ARM-1 were: sense 5'-ggacagcttggccctctcat-3'; antisense 5'-gggcaaatcacaatcaccactac-3'. Reactions for GAPDH were performed with the same primers used in semiquantitative PCR. Real time-PCR reactions were cycled 35 times through 30 s at 95°C, 30 s at 60°C, 60 s at 72°C, and 5 s at temperature of fluorescence acquisition (FA). Data were analyzed with the GeneAmp 5700 SDS software version 1.3 (Applied Biosystems).

### Analysis of FAK phosphorylation

Lysates from adhered cells were obtained with cold lysis buffer containing phosphatase inhibitors [1 mM sodium pyrophosphate; 1 mM sodium orthovanadate; 50 mM sodium fluoride]. Lysates were immunoprecipitated with anti-FAK antibody bound to protein A- agarose beads. The immunoprecipitated proteins were recovered adding SDS sample buffer, separated by SDS-PAGE, transblotted onto PVDF membranes, and probed with anti-phosphotyrosine and anti-paxillin antibodies. The same membranes were reprobed with anti-FAK antibody.

### Affinity precipitation of activated Rac-1

Pull-down assays for Rac-1 were performed using EZ-Detect activation kit from Pierce (Rockford, IL, USA), according to the manufacturer's instructions. Briefly, 10^6 ^cells were lysed in 400 μl of cold lysis buffer as described above. The samples were mixed with GST-PAK1-PBD, loaded on SwellGel immobilized glutathione disks, and incubated with constant agitation at 4°C for 2 h. Then, the disks were washed and the glutathione-bound Rac-1 (active form) was recovered adding SDS sample buffer [60 mM Tris-HCl (pH 6.8); 2% SDS; 0.05% 2-mercaptoethanol; 1% glycerol; and 0.05% bromophenol blue]. The isolated proteins as well as aliquots of cell lysates were separated by electrophoresis and Rac-1 was detected by immunoblotting using anti-Rac-1 monoclonal antibody.

### Neutral red assay

Cell viability was determined using the neutral red accumulation assay [30]. The cells were plated on 96-well plates and then treated as described above. After 12, 24, 36 or 48 h of exposure to 4-HC, the medium was changed and the cells were incubated for 90 min under cell culture conditions with 50 μg/ml neutral red. After this incubation the cells were fixed on the plate with an aqueous solution containing 1% formaldehyde and 1% calcium chloride and then lysed with 50% ethanol/49% water/1% acetic acid. The concentration of accumulated neutral red as a marker for cell viability was measured spectrophotometrically at 560 nm.

### Clonogenic assay

Cell survival was evaluated accordingly to the methodology reported by Franken *et. al*. [31]. Briefly, cells were harvested and counted after treatment. Cell suspensions from each treatment were diluted in RPMI-1640 with 5% FBS, and immediately re-plated in 6-well plates at a density of 20 cells/cm^2^. The plates were incubated until cells in control wells have formed sufficiently large colonies. After that, the colonies were fixed with 6% glutaraldehyde and stained with 0.5% crystal violet. The plates were photographed and their digital images were analyzed with ImageJ NIH software [27] to known the colony number.

### Experimental metastasis assay

B16-F10 cells were treated *in vitro*, as mentioned under "cell culture and treatments". After treatment, cells were detached with a non-enzymatic cell dissociation buffer [4 mM EDTA in Ca^2+ ^and Mg^2+^-free PBS], resuspended in Hank's balanced salt solution and immediately injected into the tail vein of 8-week old, male C57BL/6 mice. Each mouse received 10^6 ^cells. At 2 weeks after i.v. injection mice were euthanized, lungs were excised and the metastatic tumors counted in a blind manner under a dissecting microscopy.

### Differential display reverse transcription polymerase chain reaction (DD-RT-PCR)

Total RNA, free of DNA contamination was used for the DD-RT-PCR as described by Liang and Pardee [32]. Briefly, cDNA was synthesized with 200 ng of RNA and 1 μM of primer of sequence 5'-t_12_ca-3' to anneal. A control reaction lacking reverse transcriptase was included to confirm absence of non-specific amplification from genomic DNA. The cDNAs corresponding to 20 ng of RNA were PCR amplified in the presence of 0.4 μM of [α-^35^S] dATP (37 GBq/pmol) using primers of sequence 5'-t_12_ca-3' (1 μM) and 5'-gttgcgatcc-3' (0.2 μM). PCR reactions were cycled 35 times through 50 s at 95°C, 90 s at 40°C, and 60 s at 72°C. The heat denatured PCR products were electrophoresed on a urea-PAGE gel (48% urea and 6% acrylamide). The gels were dried and exposed to X-ray films at -70°C. After 10–12 h the X-ray film was developed until the DNA bands were clearly seen on the film. The DNA bands which were differentially displayed in the autoradiograph were visually selected, marked on the gel and the band was cut with a sterile razor. The DNA extracted from the gel was PCR amplified using the same set of primers used in the DD-RT-PCR analysis, cloned, and sequenced.

### Adhesion of melanoma cells to lung sections

To evaluate the organ-specific adhesion of tumor cells *in vitro*, the method of Vink *et. al*. [33] was used with modifications in incubation times. Briefly, fresh lungs obtained from healthy C57BL/6 mice were embedded in TissueTeK O.C.T compound (Poly-Labo, Strasbourg, France) and frozen at -196°C. Fresh cryostat sections (8–10 μm thick) mounted on glass slides were first incubated with PBS/BSA 3% for 60 min in a humid chamber. After treatment, melanoma cells were detached with a non-enzymatic cell dissociation buffer, resuspended in medium, and placed on tissue sections for 30 min at 37°C with gentle agitation. The slides were washed 3 times with medium to remove nonattached cells and then fixed in paraformaldehyde 3% in PBS. Slides were stained with hematoxiline-eosine and the numbers of cells attached to the cryostat sections were counted in 10 microscopic fields.

## Results

### 4-HC decreased the expression of paxillin, altering its cellular localization and the amount of phosphotyr^118^-paxillin

**Figure 1:**
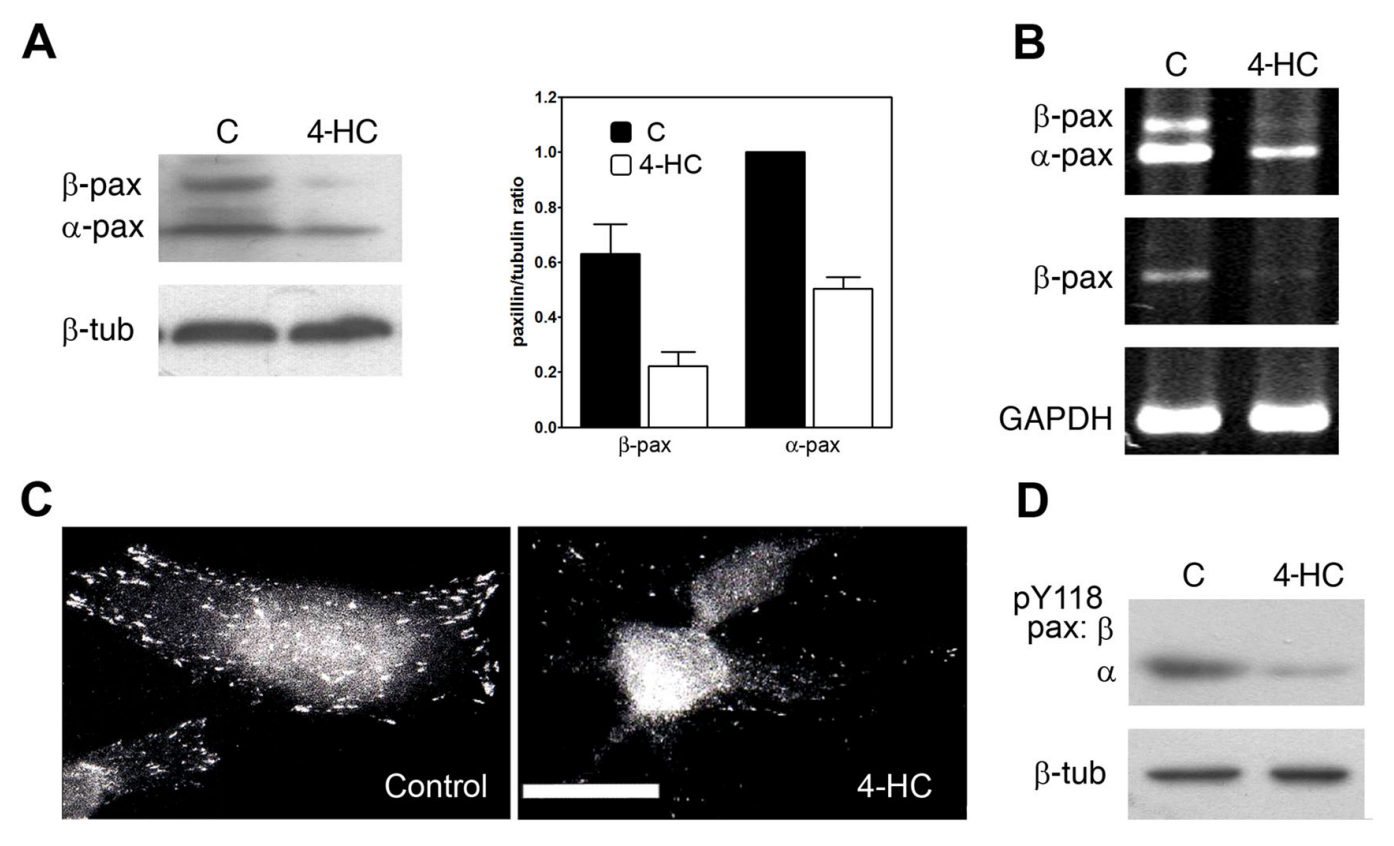
**Paxillin expression, phosphorylation and localization in 4-HC-treated cells**. *A*. Representative immunoblot showing decreased levels of both paxillin isoforms in 4-HC-treated cells. As loading control, β-tubulin was detected in the same membrane. Quantification of paxillin expression (graph) was performed by densitometric analysis from 4 independent experiments. On each experiment, the value of the band corresponding to α-paxillin in control lane was arbitrarily set to 1. Paxillin/tubulin ratios (mean ± SE) were reduced in 4-HC-treated cells (open bars in graph) when compared with control cells (closed bars). *B*. Semiquantitative RT-PCR using primers for pan-paxillin (upper panel), β-paxillin (middle panel) or GAPDH (lower panel). One representative experiment from 3 is shown. *C*. Representative photomicrographs from immunofluorescences performed with anti-paxillin antibody. In control cells (left), paxillin was located in focal adhesions around the cell periphery; this pattern was modified by the treatment with 4-HC (right). Scale bar = 20 μm. *D*. The level of phosphotyr^118^-paxillin (pY118) was also diminished by 4-HC. We were only able to detect phosphorylation in the α isofom of paxillin. One representative experiment from 3 is shown.

Analysis of paxillin expression by immunoblotting showed that B16-F10 melanoma cells normally express α and β isoforms of paxillin (Figure 1A, control lane). Treatment of cells with 4-HC decreased the expression of both paxillin isoforms. Densitometric analyses of the immunoblots revealed that in 4-HC-treated cells, the β-paxillin/tubulin and α-paxillin/tubulin ratios decreased to 35 and 50% of the control values, respectively (Figure 1A). Additionally, we performed semiquantitative RT-PCRs in order to evaluate whether the decrements of paxillin elicited by 4-HC were produced through changes in mRNA levels. Using pan-paxillin primers as well as specific β-paxillin primers we found decreased mRNA expression in cells that were treated with 4-HC (Figure 1B). Cellular localization of paxillin can regulate its activation; thus, we studied the paxillin localization through immunofluorescence assays. In control cells paxillin is distributed in a punctate pattern, presumably forming part of focal adhesions. In contrast, in B16-F10 cells treated with 4-HC paxillin localization was predominantly perinuclear (Figure 1C). As previously reported [26], 4-HC impaired the formation of lamellipodia and induced shrinkage of the outer envelope, forming a round cell with radial filopodia attached to adhesion points. Paxillin can be phosphorylated on several tyrosines [34]; among them, tyrosines 31 and 118 have shown importance in the generation of signals necessary for adhesion and migration [7,12]. Thus, we studied if the reduction in paxillin expression produced by 4-HC correlated with changes in the level of phosphotyr^118^-paxillin. Even when both isoforms can be phosphorylated, we were able to detect phosphotyr^118 ^only in the α isofom of paxillin (Figure 1D). For this isoform, the phosphotyr^118^-paxillin/paxillin ratio is 85% of the control value, indicating that the reduction in phospho-paxillin is mainly caused by paxillin downregulation.

### Paxillin-mediated signaling was impaired in 4-HC-treated cells

**Figure 2:**
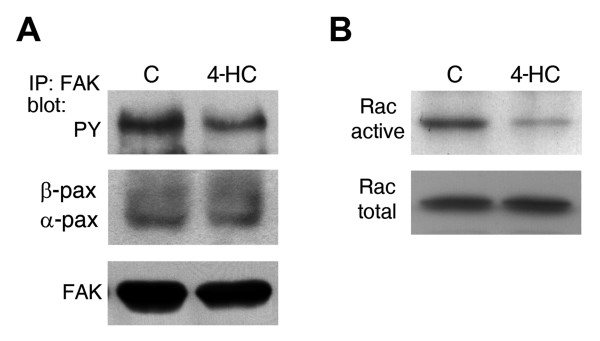
**Effect of 4-HC on the basal activation of FAK and Rac-1**. *A*. Analysis of samples immunoprecipitated with anti-FAK antibody showed that basal tyrosine phosphorylation of FAK (upper panel) was decreased by 4-HC. The same membrane was stripped and reprobed with anti-paxillin and anti-FAK antibodies. We found no changes on the FAK-bound paxillin levels (middle panel). One representative experiment from 3 is shown. *B*. The active form of Rac-1 was purified using pull-down assays and then probed with anti-Rac-1 antibody (upper panel). Detection of Rac-1 from total lysates (lower panel) showed that the difference in the amount of active Rac-1 is not associated to changes on its expression. One representative experiment from 2 is shown.

Paxillin participates in the regulation of different signaling pathways. We studied the activation of FAK, a key partner in paxillin-meditated signaling. Basal tyrosine phosphorylation of FAK was found in serum starved B16-F10, as previously reported [35] (Figure 2A, control lane). In 4-HC-treated cells, the phospho-FAK/FAK ratio decreased to 46% of the control value (Figure 2A). However, levels of FAK-bound paxillin did not change significantly (Figure 2A) suggesting that even when paxillin is downregulated, the remaining paxillin supports FAK binding.

The activation of Rac-1, which promotes the formation of lamellipodia, can be triggered by tyrosine-phosphorylated paxillin; therefore, we studied the activation of Rac-1 performing pull-down assays. 4-HC decreased the amount of GTP-bound Rac-1 without changing Rac-1 expression (Figure 2B), reducing the active Rac-1/total Rac-1 ratio to 35% of the control value.

### 4-HC did not alter proliferation or survival of B16-F10 cells

**Figure 3:**
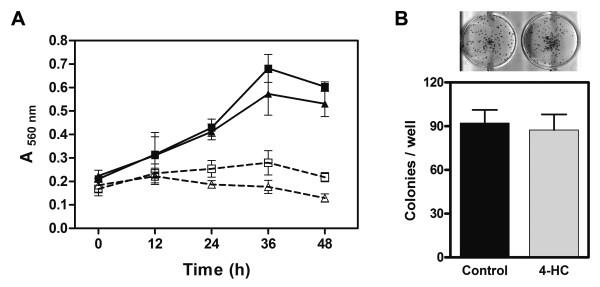
**Effect of 4-HC on cell proliferation and survival**. *A*. B16-F10 cells were treated with 500 μM 4-HC (triangles) or 0.75% ethanol (control cells; squares) and viable cells were quantified every 12 h using the neutral red accumulation assay. Experiments performed in presence of 10% FBS (solid lines) showed that cell proliferation was unaffected by 4-HC (P > 0.05, ANOVA). When cells were treated in serum-free medium (dashed lines), the initial cell number was unaltered neither by time nor by 4-HC. Values are mean ± SE obtained from 3 independent experiments. B. Clonogenic assay were performed by seeding 4-HC-treated cells at a density of 20 cells/cm^2^. After incubation for 8 d, the generated colonies were stained and counted. There was no difference in the colony number produced by 4-HC-treated cells or control cells. Values in the graph are mean ± SE obtained from 3 independent experiments. The upper picture shows examples of the results.

Reduction of paxillin expression as well as decreased FAK activation might affect cell proliferation, survival or apoptosis/anoikis [36]. For that reason, we studied the viability of cell cultures exposed to 4-HC during different times. In presence of FBS, 4-HC did not modify cell proliferation in comparison with control cells (Figure 3A, solid lines). When cultures were treated in serum-free medium, we found that 4-HC caused a slight, non significant reduction on the number of viable cells at times longer than 24 h (Figure 3A, dashed lines). Congruously, 4-HC did not affect the ability of single cells to grow into a colonies (Figure 3B), suggesting that cell survival is unaffected by 4-HC.

### Treatment of B16-F10 cells with 4-HC decreased their ability to form experimental metastases

**Figure 4:**
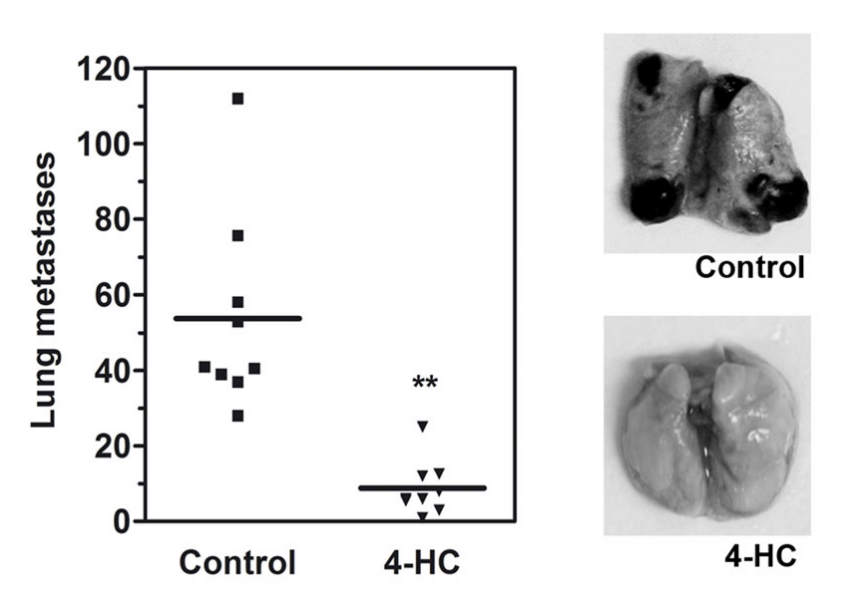
**Effect of 4-HC on the formation of experimental metastases**. Melanoma cells were treated *in vitro *with 500 μM 4-HC for 24 h, and then injected into the tail vein of C57BL/6 mice. Two weeks later, mice were sacrificed and the macroscopic pulmonary tumors were counted. Mice injected with 4-HC-treated cells (triangles) showed a 7-fold decrease in the number of lung tumors compared with the mice that received control B16-F10 cells (squares). ** P = 0.002 (Student's *t *test with Welch's correction). The pictures on the right show examples of lungs from mice injected with either control or 4-HC-treated cells.

In order to analyze the relevance of the effects produced by 4-HC in metastasis, we evaluated the effect of 4-HC in an experimental metastasis model. In that model, B16-F10 cells previously treated *in vitro *were injected into the tail vein of C57BL/6 mice. When control cells were introduced, they metastasize to the lungs and form 55.5 ± 10.1 (mean ± SE; n = 9) black spherical colonies. In contrast, 4-HC-treated cells formed only 8.8 ± 2.4 (mean ± SE; n = 9) of such colonies (Figure 4). Data were analyzed with computer-assisted *t *test and the difference was highly significant.

### Paxillin downregulation by siRNA diminished the number of experimental metastases

**Figure 5:**
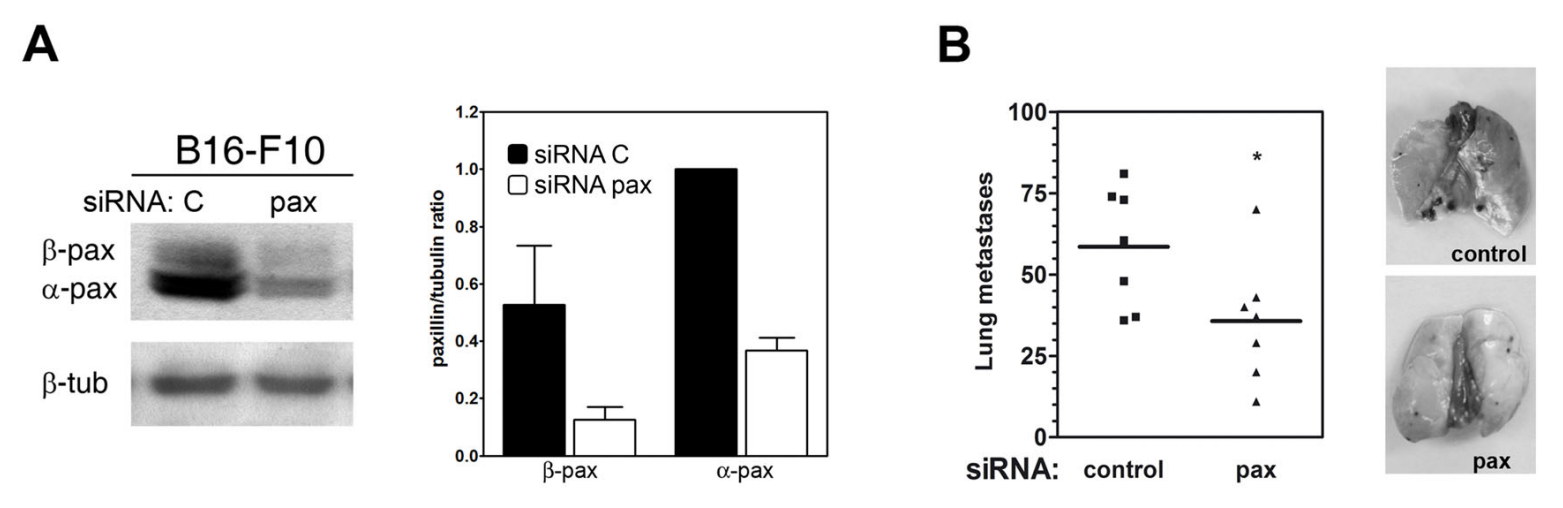
**Effect of paxillin-siRNA on the formation of experimental metastases**. *A*. Immunoblot showing that paxillin expression was reduced in B16-F10 cells transfected with paxillin-siRNA (pax). Densitometric quantification (graph) show that the decrements on paxillin/tubulin ratios (mean ± SE, n = 3) in cells transfected with paxillin-siRNA (open bars) were similar to those produced by 4-HC. *B*. The participation of paxillin in metastasis formation was studied by injecting into mice cells transfected with either paxillin- or control-siRNA. Paxillin downregulation produced a 1.6-fold reduction in the number of experimental pulmonary metastases. * P = 0.04 (Student's *t *test). The pictures on the right show examples of lungs from mice injected with siRNAs-trated cells.

In order to study if paxillin inhibition was enough to block the formation of metastasis, we analyzed the metastatic potential of B16-F10 cells transfected with paxillin-siRNA. These cells showed reduced expression of both paxillin isoforms to levels similar of those found in 4-HC-treated cells (Figure 5A). Employing the model described above, we found that melanoma cells transfected with the control-siRNA formed 58.5 ± 7.0 (mean ± SE; n = 7) pulmonary tumors. When cells transfected with paxillin-siRNA were injected, the number of tumors was modest but significantly reduced to 35.7 ± 7.1 (mean ± SE; n = 7) (Figure 5B).

### Differential display RT-PCR of cells treated with 4-HC

To evaluate other changes in gene expression in melanoma cells treated with 4-HC, we employed the differential display RT-PCR analysis. Several transcripts were differentially displayed between control and 4-HC-treated cells (data not shown). One downregulated band was purified, reamplified, cloned, sequenced, and compared to the nucleotide database in GenBank. The transcript was identified as Adhesion Regulating Molecule-1 (ARM-1; GeneBank accession: NM_019822).

### 4-HC diminished ARM-1 expression and impaired adhesion to lung slides

**Figure 6:**
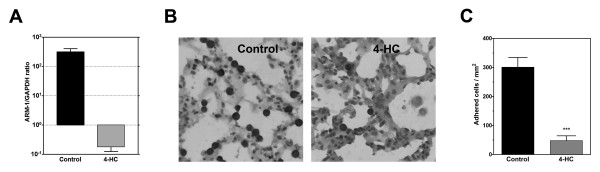
**ARM-1 expression and adhesion to lung sections by cells treated with 4-HC**. *A*. The effect of 4-HC on ARM-1 expression was evaluated by real time-PCR. 4-HC inhibited ARM-1 mRNA expression. Values are represented as ratios of ARM-1/GAPDH ± SD. *B*. B16-F10 cells were treated as described previously, and then incubated with lung sections during 30 min to allow their adhesion. The preparations were stained and photographed. Photomicrographs (400X) show that control cells (left) were able to adhere to lung sections; in contrast, 4-HC-treated cells (right) displayed impaired adhesion. *C*. Quantification of adhered cells (mean ± SE) from 5 independent experiments. On each experiment, 10 fields were counted. ***P < 0.0001 (Student's *t *test).

Downregulation of ARM-1 was confirmed by real time RT-PCR. In 4-HC-treated cells, ARM-1/GAPDH ratio was diminished 1800-fold (Figure 6A). ARM-1 expression promote adhesion to endothelial cells [37]; therefore, we studied the effect of 4-HC on the adhesion of melanoma cells to lung sections. Our results showed that 4-HC produced a 7-fold reduction in the number of adhered cells (Figures 6B and 6C), suggesting that ARM-1 downregulation may participate in the antimetastatic effect of 4-HC.

## Discussion

Paxillin is involved in the regulation of different cellular functions such as modulation of cytoskeletal organization, adhesion, and motility [7-12]. Therefore, paxillin expression and phosphorylation are important in the acquisition of an invasive behavior. For example, paxillin is overexpressed and hyperphosphorylated in sublines of the osteosarcoma cell line HuO9 that are highly metastatic, compared with the low-metastatic sublines [22]. The present study demonstrates that 4-HC decreases the expression of both α- and β-paxillin isoforms at a transcriptional level in B16-F10 cells. Paxillin downregulation correlates with an inadequate translocation of paxillin to focal adhesions, a decreased pool of phosphotyr^118^-paxillin, and alterations on paxillin-mediated signaling pathways.

The basal phosphorylation of FAK is reduced by 4-HC. Fully activation of FAK needs its translocation to focal adhesions where can be hyperphosphorylated by Src [38]. Once activated, FAK can promote cell migration through multiple signaling connections [39]. Accordingly, increased expression of FAK has been found in numerous neoplasms including melanoma where correlates with increased cell motility [40] and a more aggressive phenotype [41]. Paxillin binding to FAK is partially responsible of FAK translocation to focal adhesions [42,43]. The interaction between paxillin and FAK also promotes the tyrosine phosphorylation of paxillin [44], which in turn may alter paxillin binding affinity to FAK [42]. Thus, the formation of a FAK-paxillin complex is involved in the regulation of dynamics of both proteins and in the activation of signaling pathways required for migration. We found that 4-HC reduced the basal phosphorylation of FAK without altering the paxillin binding to FAK. This suggests that paxillin downregulation plays a minor role in reduced FAK phosphorylation. Accordingly, paxillin ^-^/^- ^cells show only a small but consistent decrement in phosphorylated FAK [45]. On the other hand, the localization of paxillin at focal adhesions is highly dependent on the integrity and dynamics of actin networks [46]. In endothelial cells treated with cytochalasin D to disrupt actin microfilaments, paxillin almost totally disappear from focal adhesions [46]. Since the formation of stress fibers is inhibited by 4-HC [26], we hypothesize that reductions in activated FAK and tyrosine-phosphorylated paxillin are caused by a restrain of paxillin-FAK complex to reach focal adhesions.

4-HC also decreased the basal activation of Rac-1, a downstream effector of paxillin, indicating that reduced paxillin expression affects this pathway involved in the regulation of motility. The participation of tyrosine-phosphorylated paxillin in Rac-1 activation is well documented [47,48]. The phosphorylation of tyrosines 31 and 118 on paxillin generate binding sites for the adaptor protein Crk [47]. Crk-paxillin interaction promotes the binding of DOCK180 to Crk, which in turn locally activates Rac-1 [48]. Once activated, Rac-1 has a key role in cell motility through its ability to stimulate lamellipodium protrusion at the leading edge [4,49]. Then, the reduced Rac-1 activation in 4-HC-treated cells, which correlates with the lack of lamellipodia previously reported [26], seems to be subsequent to the reduced phospho-paxillin level. Notoriously, basal activation of Rac-1 is increased in B16-F10 cells relative to the poorly metastatic B16-F0 cells [50]; therefore, impaired Rac-1 activation by 4-HC may be cooperating to decrease the metastatic behavior of B16-F10 cells.

Changes in paxillin expression or FAK activation can promote alterations on cell proliferation and survival [36,39]. In this study we found that exposition to 4-HC during 48 h did not modify cell proliferation, indicating that in B16-F10 cells this process is unaffected by paxillin downregulation. Cell survival was also unaltered by 4-HC, demonstrating that the antimetastatic effect of 4-HC is not related to changes in long term survival of melanoma cells. The lack of cytotoxic effect of 4-HC on cancer cells reported here is consistent with previous studies [26,51]. Furthermore, 4-HC (500 μM) does not affect the viability or the cytoskeleton stability of B82 fibroblasts [26] and is nontoxic to cultured hepatocytes [52], supporting the hypothesis that 4-HC has low toxicity.

Additionally, our results show that paxillin downregulation, either by 4-HC or paxillin-siRNA, reduce the metastatic potential of B16-F10 cells. Contrary to other molecules involved in the regulation of adhesion, such as integrins or FAK, the role of paxillin in the biology of metastasis is still controversial. Several reports identify paxillin as an inducer of metastasis; for instance, in head and neck cancers that have metastasized to lymph nodes paxillin expression is increased [53]. Similarly, paxillin up-regulation correlates with the presence of extrahepatic metastasis in hepatocellular carcinoma [54] and lymph node metastasis in breast tumors [55]. On the contrary, other reports state that paxillin overexpression is a marker of a less invasive tumor phenotype in breast [56] and lung [57] carcinomas. The fact that metastatic potential of B16-F10 cells is reduced by decreasing paxillin expression support the hypothesis that paxillin facilitates metastasis and emphasize the importance of paxillin as an inducer of melanoma metastasis. Nevertheless, the number of pulmonary metastases produced by paxillin-silenced cells is 4-fold greater than the quantity produced by 4-HC-treated cells, indicating that paxillin downregulation is only partially responsible for the antimetastatic effect of 4-HC.

To further understand the mechanism involved in the 4-HC antimetastatic effect, we carried out a differential display RT-PCR assay and discovered that 4-HC may be altering the expression of several genes other than paxillin. In this work ARM-1 has been identified as one of the genes differentially expressed in 4-HC-treated cells. ARM-1 is a protein associated with the plasma membrane [58,59] whose precise functions have not been totally solved. ARM-1 was first discovered as an adhesion promoting molecule [37,59], but recently it was identified as a novel component of the 26 S proteasome [60]. The role of ARM-1 in the regulation of cell adhesion is supported by studies in which changes in ARM-1 expression alter cell-cell adhesion. For example, overexpression of ARM-1 in endothelial cells increases lymphocyte adhesion [58]. Similarly, transfection of ARM-1 into kidney embryonic cells promotes their adhesion to endothelial cells [37]. A role of ARM-1 in metastasis is suggested by the fact that ARM-1 expression is increased in metastatic human breast cancer cells as compared with their non metastatic counterparts [37]. Besides, ARM-1 expression is constitutive in cell lines from gastric carcinoma [59], breast carcinoma, and T lymphoma [37]. Together, these data suggest that ARM-1 may be a metastasis-associated gene. We show that ARM-1 dowregulation produced by 4-HC correlates with a decreased adhesion of B16-F10 cells to lung sections. This effect suggests that 4-HC may be inhibiting ARM-1-mediated tumor cell adhesion and/or invasion during the formation of pulmonary metastases. Nevertheless, the role of ARM-1 reduced expression in the antimetastatic effect of 4-HC needs further investigation. Furthermore, since several transcripts were differentially displayed in 4-HC-treated cells, it cannot be ruled out that 4-HC may be affecting the expression of other regulatory proteins that participate in metastasis. In particular, it will be necessary to clarify the effect of 4-HC on integrin expression since several of these receptors participate in different stages of the metastatic cascade.

## Conclusion

This study shows that 4-HC inhibits the formation of experimental metastases. Although the molecular mechanism of the antimetastatic action of 4-HC needs to be further defined, our present results indicate that 4-HC-induced decreased paxillin availability and inappropriate subcellular localization impair the adequate generation of signals that promote malignancy. In addition, 4-HC may affect important steps of metastasis by changing the expression of other molecules, such as ARM-1. However, further studies are required to clarify the effects of 4-HC *in vivo *since there are not toxicological or pharmacokinetic data available. Together, our results suggests that 4-HC, or agents with similar mode of action, might be useful in the prevention of metastasis and could be used as an adjuvant in the therapy of melanoma.

## Competing interests

The authors declare that they have no competing interests.

## Authors' contributions

MAVV performed cell transfections and clonogenic assays, helped to carry out FAK and Rac1 activation assays, designed and supervised the study, and prepared the manuscript.

NSJ performed cell culture and carried out immunoblots, RT-PCRs, immunofluorescences, and experimental metastases assays.

NMP participated in the design of the study.

JJM participated in the design of the study and in the preparation of the manuscript.

All authors read and approved the manuscript.
